# Limitations of a polymer-based hole transporting layer for application in planar inverted perovskite solar cells†

**DOI:** 10.1039/c9na00246d

**Published:** 2019-06-21

**Authors:** Miloš Petrović, Temur Maksudov, Apostolos Panagiotopoulos, Efthymis Serpetzoglou, Ioannis Konidakis, Minas M. Stylianakis, Emmanuel Stratakis, Emmanuel Kymakis

**Affiliations:** Department of Electrical & Computer Engineering, Hellenic Mediterranean University Heraklion 71410 Crete Greece kymakis@staff.hmu.gr; Department of Materials Science and Technology, University of Crete Heraklion 71003 Crete Greece; Physics Department, University of Crete 71003 Heraklion Crete Greece; Institute of Electronic Structure and Laser (IESL), Foundation for Research and Technology–Hellas (FORTH) 71110 Heraklion Crete Greece

## Abstract

Planar inverted lead halide photovoltaics demonstrate remarkable photoconversion properties when employing poly(triarylamine) (PTAA) as a hole transporting layer. Herein, we elucidate the effect of ambient ultraviolet (UV) degradation on the structural and operational stability of the PTAA hole transporter through a series of rigorous optoelectrical characterization protocols. Due attention was given to the interplay between the polymer and perovskite absorber, both within the framework of a bilayer structure and fully assembled solar cells. The obtained results imply that UV degradation exerts a major influence on the structural integrity of PTAA, rather than on the interface with the perovskite light harvester. Moreover, UV exposure induced more adverse effects on tested samples than environmental humidity and oxygen, contributing more to the overall reduction of charge extraction properties of PTAA, as well as increased defect population upon prolonged UV exposure.

## Abstract 2

Operational stability and structural integrity of a poly(triarylamine) hole transporter and methylammonium lead halide absorber are investigated upon exposure to UV stress.

## Graphical abstract

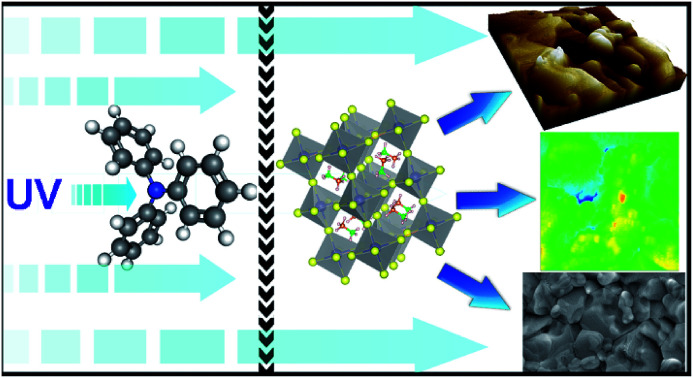

## Introduction

With the ever-increasing interest of the scientific community in the prospective applications of large area perovskite photovoltaics pertaining to industry-scale fabrication, recent research trends have been focused on increment of device operational stability. Resolution of such an important issue would further expedite the adoption of lead halide solar cells for large-scale applications, which is further supported by energy conserving, cost effective and high through-output solution processing. Most of such efforts were aimed towards application of devices built upon mesoporous architectures, as they exhibited the highest power conversion efficiency (PCE) and operational stability. Such a structure, referred to as regular or normal, commonly utilizes TiO_2_ as an electron transporting layer (ETL) and various materials as hole transporting layers (HTLs)^1–5^ Usually, HTL materials containing carbocyclic cores rely on the presence of π-conjugated bonds to shield electroactive fractions of crosslinked chains.^6^ Additionally, terminal alkoxy groups present in such systems promote solubility and open up possibilities for adjustment of the highest occupied molecular orbitals,^7^ by aligning their energy level with the valence band of the perovskite absorber. Charge transport can be further improved by using HTL materials containing cyclic hydrocarbon scaffolds built around silicone cores^8^ or *via* substitution of carbon atoms within the hydrocarbon ring with sulphur. Subdivisions formed in such a way provide additional electron pairs, aiding charge extraction through π–π stacking.^9^ However, when keeping in mind more simplistic and energy efficient fabrication processes suitable for low temperature processing conditions, an inverted planar structure (HTL/perovskite/ETL) is suggested as a promising alternative to mesoporous cells. In essence, adoption of inverted device architectures opens the door to implementation of cheaper flexible substrates and further reduction of overall manufacturing costs. Unlike their inorganic counterparts, organic materials have an intrinsic benefit of lower processing temperatures and PTAA stands out as the suitable and the most popular candidate for the HTL material in inverted planar perovskite solar cells. The structure of PTAA is built upon successive repetition of triarylamine units forming a high molecular weight polymer network and providing good substrate coverage, which in turn promotes nucleation of perovskite crystals, resulting in larger grains.^10^ The reasons for the selection of PTAA are numerous, but the most important ones are resilience to negative effects of oxygen and moisture and remarkable intrinsic hole mobility (∼4 × 10^−3^ cm^2^ V s^−1^),^11^ which can be further improved by doping, thanks to available nitrogen electron pairs located at triphenylamine bonding sites.^12,13^ Numerous attempts were made to improve the stability of devices employing PTAA, mainly *via* doping or application of interfacial barriers to prevent ion migration from the light harvester and extend operational lifetime.^14–18^ Although satisfactory improvements were suggested by many groups, there is surprisingly a low number of studies concerning the stability of PTAA itself. Namely, literature reports on this topic are usually related to PTAA stability after heat and moisture induced oxidation.^19–21^ To the best of our knowledge, there are no reports elucidating the behaviour of PTAA upon prolonged exposure to UV irradiation, which is of crucial importance for estimation of long-term stability for perovskite based photovoltaics. The effect of ultraviolet (UV) exposure on degradation of devices with mesoporous structures is associated with photocatalytic behaviour of TiO_2_ and the resulting interplay between accumulated defects at and disruption of charge transfer across the interface with the perovskite.^22,23^ Despite the aforementioned research progress related to inverted planar perovskite cells and remarkable PCEs achieved with this structure,^1^ there is a notable lack of information regarding the impact caused by UV induced degradation on the operational and structural stability of devices utilizing PTAA as a HTL. Our work aims to bridge this gap, by presenting readers with comprehensive and detailed studies on the effect of short wavelength photons on the stability and charge transport of perovskite solar cells containing PTAA as a hole transporter, while taking into consideration the interactions at the PTAA/MAPI interface, as well as the overall photophysics of fully assembled devices. We conducted a series of optoelectrical spectroscopy and space-charge limited experiments to monitor the effect of UV degradation on neat PTAA films, bilayers with methylammonium lead iodide light harvester (MAPI), and finally, on fully assembled solar cells with an inverted planar structure (ITO/PTAA/MAPI/PC_60_BM/PFN/Ag). Since UV radiation accounts for approximately 5% (4.6 mW cm^−2^)^23^ of the AM1.5G spectrum, we opted for even harsher experimental conditions by exposing our samples to more than a double of that intensity, totalling to 10 mW cm^−2^. In this way, we ensured that there was no underestimation of the UV impact encountered during the operation of solar cells under “real-life” conditions. In principle, the presence of encapsulant foil prevents transmission of high energy photons and provides a sufficient level of protection from UV irradiation during outdoor operation. However, the ageing process may lead towards undesirable effects such as yellowing and delamination of the protective barrier, resulting in device exposure to UV stress accompanied by oxygen and moisture ingress. In order to replicate such a scenario as close as possible, we conducted all of the experiments under ambient conditions, ensuring that all three adverse factors are accounted for. In such a way, we exclude any possible underestimation of the degradation degree arising from the isolated effect of UV, since such a degradation process would also involve the presence of oxygen/moisture under realistic operational circumstances. The effect of UV degradation on prepared samples was observed at various exposure steps (2, 5, 8, 12, 16 and 24 hours) and the results at each stage of degradation were used to quantify the divergence from the behaviour of a control device stored under ambient dark conditions. Interfacial behaviour was probed with ultrafast transient absorption spectroscopy (TAS)^24^ and small/large perturbation transient photovoltage/photocurrent (TPV/TPC) measurements.^25,26^ Population of defect states and interplay with drift mobility of charge carriers were observed using space-charge-limited-current (SCLC)^27^ and photoinduced charge extraction by linearly increasing voltage (photo-CELIV).^28^ In this way, reliable assessment of charge transport properties recorded at various steps of device assembly and stages of degradation allows elucidation of device behaviour covering both short (femtosecond TAS) and longer timescales (TPV/TPC, SCLC, photo-CELIV). At all stages of experiments, the structural integrity of both MAPI and PTAA was monitored and appropriate connection with charge transport properties was drawn.

## Materials and methods

### Device fabrication

Indium tin oxide (ITO) prepatterned substrates (20 × 15 mm, Naranjo Substrates) with a sheet resistance of ∼15 Ω sq^−1^ were cleaned in a three-step process consisting of ultrasonication with deionized water, acetone and isopropanol for 10 minutes each. After drying, the substrates were transferred to a nitrogen filled glovebox for ozone treatment, in order to remove any organic residues. A hole transporting layer was spin-coated from a solution of PTAA (12 mg ml^−1^, Ossila, 28 422 kDa) in toluene at 6000 rpm, followed by annealing at 110 °C for 10 minutes. Afterwards, MAPI was fabricated according to the Lewis acid/base adduct protocol described by Lee *et al.*^29^ from the precursor solution 1 mmol of PbI_2_ (Sigma-Aldrich), 1 mmol of methylammonium iodide (MAI, Dyesol), 0.9 mmol of dimethyl sulfoxide (DMSO, Sigma-Aldrich) and addition of thiourea (0.1 mmol, Sigma-Aldrich) in 636 μl of anhydrous *N*,*N*-dimethylformamide (Sigma-Aldrich). Perovskite layers were spun over the previously prepared polymer film at 6000 rpm for a total of 30 seconds and anhydrous diethyl ether antisolvent was added 10 seconds into the spinning step. Thereafter, the MAPI films were annealed at 100 °C for 10 minutes and the bilayer samples obtained in such a way (ITO/PTAA/MAPI) were used for TAS measurements. On the other hand, devices intended for transient photovoltage/photocurrent characterization were fabricated by the addition of a 100 nm thick silver electrode over the bilayer, resulting in an ITO/PTAA/MAPI/Ag structure. Next, hole-only devices used during SCLC experiments were formed by evaporation of an 80 nm thick Au electrode over the previously described bilayer to yield a symmetric ITO/PTAA/MAPI/Au structure. Finally, fully assembled solar cells employed in steady state *J*–*V* and drift mobility investigations (photo-CELIV) were fabricated after PC_60_BM (Solenne BV) was spin-coated over the bilayer (20 mg ml^−1^ in anhydrous chlorobenzene) at 1500 rpm followed by deposition of poly[(9,9-bis(3′-(*N*,*N*-dimethylamino)propyl)-2,7-fluorene)-*alt*-2,7-(9,9-dioctylfluorene)] (PFN) (Solaris Chem, 0.4 mg ml^−1^) in anhydrous methanol. Afterwards, 100 nm of Ag was thermally evaporated over a shadow mask to outline an active area of 4 mm^2^ for a completely assembled planar inverted cell architecture (ITO/PTAA/MAPI/PC_60_BM/PFN/Ag).

### Characterization

UV degradation was conducted within a custom-built chamber illuminated with a series of PHILIPS UV-A lamps emitting at 350–400 nm (distribution peak at 370 nm) totalling to 10 mW cm^−2^. The degradation process was monitored under the ambient atmosphere in the absence of external light and relative humidity was kept close to 45%. The power conversion efficiency (PCE) of the fabricated solar cells was measured under a N_2_ atmosphere and 100 mW cm^−2^ illumination generated by a Xenon lamp calibrated with an Oriel 91150v silicon solar cell (Newport). Steady-state *J*–*V* curves were recorded at a constant scan rate of 10 mV s^−1^ using a multiplexer test board (Ossila) and accompanying software suite. External quantum efficiency (EQE) spectra were recorded using an integrated (QE-R3011, Enlitech) system and a chopper operating at a frequency of 146 Hz. Scanning electron microscopy images were taken using a Jeol JSM-7000F operating at 15 kV. The topography of samples was examined with an atomic force microscope (AFM) (XE7, Park Systems) operating in tapping mode, with the addition of an external lock-in amplifier (Stanford Research Systems, SR830 DRP) for a Kelvin probe force microscopy (KPFM) configuration. Calibration of the cantilever (NCSTAu, Park Systems) for work function measurements was done using a standard HOPG sample. X-ray diffraction (XRD) patterns were obtained using a D/MAX-2000 X-ray diffractometer with monochromatic Cu Kα irradiation (*λ* = 1.5418 Å) at a scan rate of 4° min^−1^. Absorbance of PTAA/MAPI bilayers was measured in the 250–850 nm range, using a UV-vis spectrophotometer (Shimadzu, UV-2401 PC). Attenuated Total Reflection (ATR) IR spectra were recorded on a Bruker Vertex 70v spectrometer. Transient absorption spectroscopy measurements were performed on a Newport (TAS-1) transient absorption spectrometer. The excitation source was a pulsed Yb:KGW-based laser (PHAROS, Light Conversion) with the central wavelength at 1026 nm, a pulse duration of 170 fs and a repetition rate of 1 KHz. During the study, the pump fluence was kept constant at 1.5 mJ cm^−2^. Optoelectrical characterization was performed on a transient module of a commercially available measurement platform, ARKEO (Cicci Research s.r.l.). Transient photovoltage was operated in the small-perturbation regime by confining the amplitude of the LED pulse to <10% of the equilibrium voltage established at the background field. This approach ensured that the transient voltage signal followed mono-exponential decay and the resulting fits mirrored the lifetime of charge carriers. On the other hand, the transient photocurrent response was monitored under large-perturbation conditions and 50 μm extraction to an external circuit, while the duty cycle was set at 1/2 in order to allow sufficient time for device loading and relaxation of decay tails. Open circuit voltage and short circuit current conditions were controlled *via* response from transimpedance and differential voltage amplifiers. Charge injection during photo-CELIV experiments was performed using a fast LED (470 nm) with a Lambertian radiation pattern and 120° viewing angle operating at 100 mA of driving current. The offset voltage was set to prevent premature extraction of the generated charge carriers between the injection pulse and extraction ramp. The selected range (0.4–0.8 V) was conditioned with the degradation stage of measured solar cells. The data were collected using a high-speed waveform generator driving one electrode with a ramp of 1.25 × 10^5^ V s^−1^ applied after 3 μs delay, while the other electrode was connected to a transimpedance amplifier and monitored with a 100 MHz single-shot bandwidth digitizer.

## Results and discussion

**Fig. 1:**
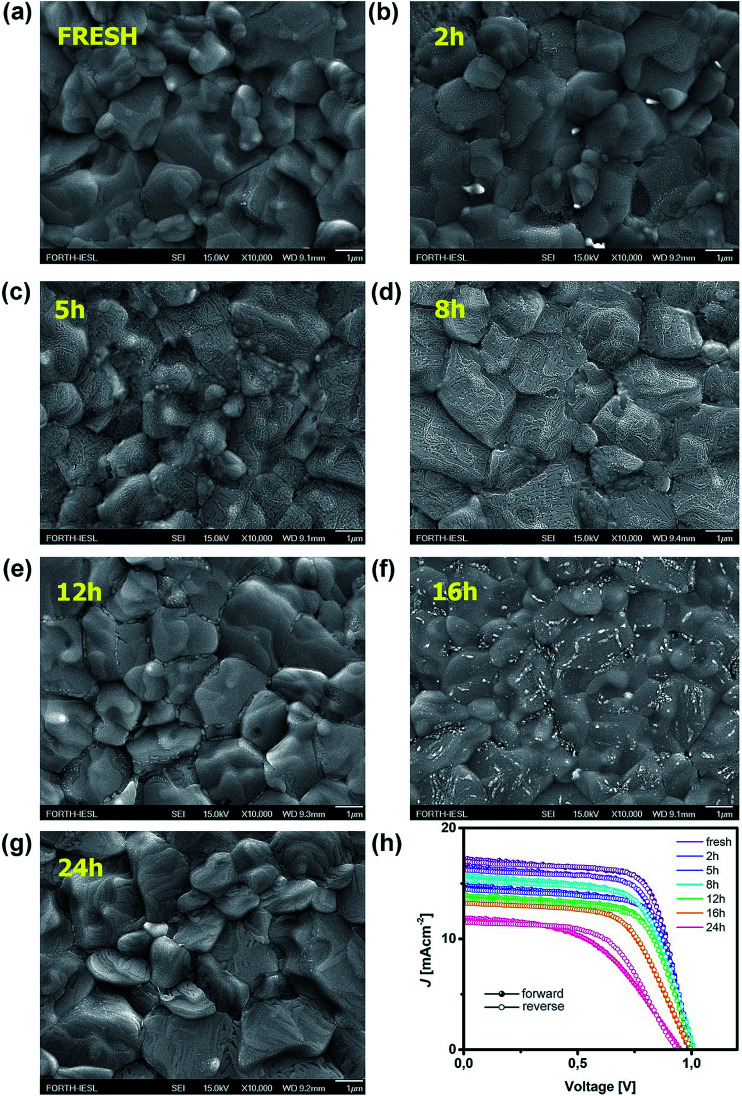
SEM images of MAPI films grown on PTAA films for (a) fresh and UV treated ITO/PTAA/MAPI bilayers after (b) 2 h, (c) 5 h, (d) 8 h, (e) 12 h, (f) 16 h and (g) 24 h of illumination; (h) *J*–*V* curves of reference and UV degraded inverted planar solar cells (ITO/PTAA/MAPI/PC_60_BM/PFN/Ag).

In this study, the effect of UV degradation was monitored within the operational framework of a bilayer structure (ITO/PTAA/MAPI) with or without a top electrode and fully assembled inverted perovskite solar cells (ITO/PTAA/MAPI/PC_60_BM/PFN/Ag) incorporating a fullerene ETL. In all cases, MAPI films were coated over 40 nm thick layers of PTAA. Bilayers formed in this way were exposed to UV radiation (illuminated from the polymer side) in the presence of oxygen and ambient humidity, spanning over a total duration of 24 hours, with sampling intervals at 2, 5, 8, 12, 16 and 24 h. Doping of MAPI solution with thiourea has been previously demonstrated as a highly reproducible fabrication method yielding large crystals, due to the Lewis acid/base interactions between electron pairs from S-donors and acceptors from lead halides.^29,30^ Consequently, we obtained perovskite grains of remarkable size, often exceeding 3 μm, as clearly demonstrated by SEM micrographs shown in Fig. 1. Moreover, MAPI crystals retained their large size irrespective of UV exposure time. It is interesting to notice the formation of narrow channels and ridges on the surface of MAPI, appearing in samples with exposure times exceeding 2 h. To further address this peculiar observation, we conducted AFM topography scans and examined the evolution of morphological alterations caused by different periods of UV degradation. The results were in line with SEM observations, as the appearance of surface irregularities became more pronounced in samples subjected to longer UV irradiation intervals and the films exhibited an overall increase of roughness (Fig. S1†). We noticed that the root mean square roughness (RMS) of the sample left for 24 h under UV light was more than double (39.14 nm) that of the fresh reference (19.19 nm). In order to exclude the effects of oxygen and moisture from the environment on the formation of recesses and ridges on the films, we aged an additional sample for 24 h under ambient conditions and in the absence of light. Indeed, no surface carvings were observed (Fig. S1†), resulting in a smoother film having an RMS of 24.98 nm. Even though the effect of oxygen on film roughness can't be completely ignored due to the significantly higher RMS than that of the reference specimen, it is safe to assume that it plays no part in the formation of a jagged surface, since the appearance of these morphological changes becomes noticeable only after 2 h of UV degradation. Thus, ultraviolet radiation appears to be the main culprit for the abovementioned surface irregularities, suggesting that the energy received through the absorption of photons emitted from the UV range affects the stability of the absorber, despite the fact that bilayers were exposed to UV light from the PTAA side. Namely, PTAA absorbs in the UV region and extended exposure leads to its partial degradation, as we will discuss in more detail later on. As illustrated in Fig. S4,† PTAA absorbs in the UV region and film deterioration reduces this intensity, which in turn decreases the shielding of the MAPI layer from the effect of UV photons. Readers should note that neat PTAA films exposed to the same UV degradation conditions did not undergo any alterations of the surface configuration (Fig. S2†), therefore the observed behaviour is most likely linked directly to the influence of UV light on the perovskite and by extension to the charge transport across the interface.

**Fig. 2:**
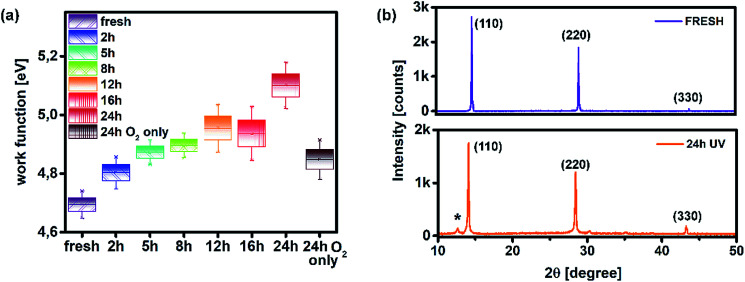
(a) Work function distribution of MAPI as a function of various ageing conditions and (b) XRD patterns of the fresh sample and that treated with UV for 24 h.

Such behaviour can be ascribed to two major factors and any combination thereof. The first one is the increased number of grain boundaries^31^ and the second is the accumulation of thiourea between MAPI grains,^32^ since an excess of Lewis base has been known to indirectly promote the degradation of MAPI in combination with its photo-inactive nature and increased number of grain boundaries. As we can see from SEM images, there is a visible transition from surface irregularities towards the formation of small grains distributed between the large crystallites, which should increase the number of boundaries and defect population, as we will describe in more detail in the section discussing the distribution of trap states. The second factor is the reception of energy from the short wavelength photons, which is large enough to kick-start the migration of halide vacancies and mobile species with low activation energies,^33–35^ leading to the degradation of the light harvester and change of the way it interacts with PTAA. Precise estimation of the extent to which each of these factors is influenced by UV degradation requires separate investigation and exceeds the scope of this study. Nonetheless, contributions of both textured grains and ion migration caused by reordering of charged species are reported to have a noticeable impact on the work function distribution.^36,37^ Thus, we utilized KPFM measurements to obtain the work function distribution of the perovskite bilayer at each stage of UV degradation, and Fig. 2a depicts the resulting surface work function spread of MAPI. It is evident that prolonged UV exposure lowers the work function of the perovskite, increases the dispersion of surface distribution and introduces more outlier values. Contribution of oxygen and humidity to this trend should not be ignored, since 24 h of ageing in air devoid of UV light caused on average 0.1 eV work function deviation from that of the reference sample. However, with the added impact of UV illumination this shift increased fourfold.

**Fig. 3:**
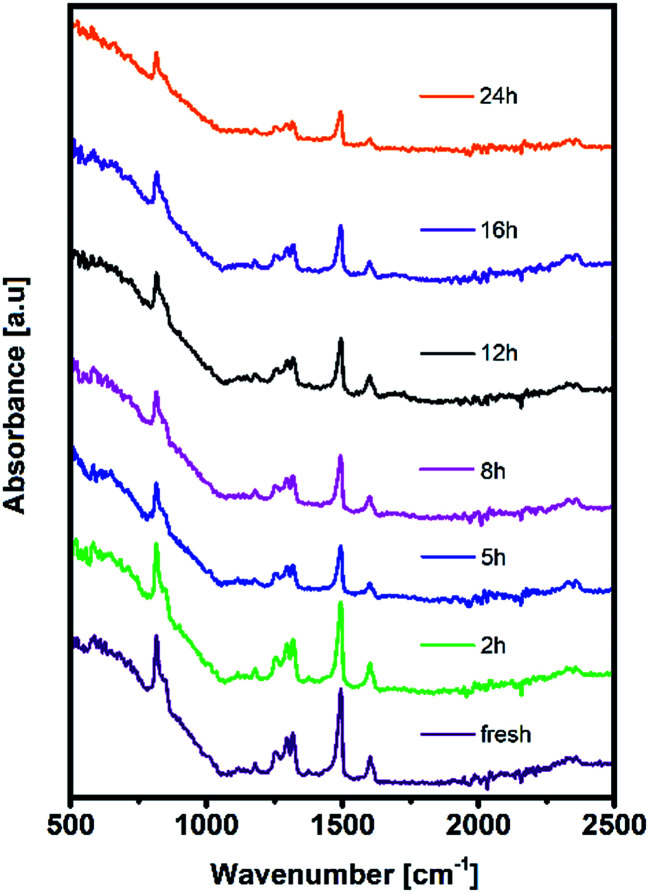
Attenuated total reflection IR spectra of fresh and UV degraded PTAA films.

Moreover, distortion of the surface potential seems to be governed by the effect of UV exposure, since the sample exposed to only oxygen and moisture yielded more uniform contrast maps, as shown in Fig. S3.† Both theoretical predictions and experimental results report the presence of iodine interstitials acting as migrating defects and playing an important role in the hysteretic behaviour of lead halide photovoltaics.^38–40^ Hence, we fabricated complete solar cells with the addition of an ETL and buffer layers over UV pre-treated bilayer samples, to obtain an inverted planar architecture (ITO/PTAA/MAPI/PC60BM/PFN/Ag). The resulting steady-state current–voltage curves shown in Fig. 1h (see the ESI† for the list of operational parameters) clearly confirm the appearance of pronounced hysteresis phenomena upon 24 h of UV stress. Further confirmation of ion migration can be obtained by comparing the XRD patterns of fresh PTAA/MAPI bilayers and those of bilayers after 24 h of UV degradation in the ambient atmosphere, as displayed in Fig. 2b. In particular, reduction of bonding strength between the methylammonium ion and inorganic cage facilitates ion migration and erodes the structure of perovskite crystals.^41,42^ This process can be identified by the emergence of an additional peak in the XRD spectrum at 12.7° originating from the presence of the PbI_2_ phase.^42–44^ Indeed, the corresponding peak marked with an asterisk appears in the pattern of the UV treated sample. It should be kept in mind that the perovskite retains its structural integrity even upon exposure to the harshest degradation conditions, as all diffraction peaks characteristic of the 110, 220 and 330 planes of MAPI remained detectable and retained high intensity.^45–47^ The degradation of PTAA films was monitored using ATR-IR spectroscopy performed under vacuum conditions in order to exclude the appearance of unwanted peaks originating from the environment. The resulting spectra recorded for each period of UV exposure are shown in Fig. 3. We notice the absence of any peaks in the 1670–1820 cm^−1^ region corresponding to the stretching of C=O bonds resulting from oxidative degradation.^19^ Therefore, deterioration of PTAA is limited to the interplay between hydrocarbon rings and polymer chain length. The declining intensity of the peak at 1493 cm^−1^ ascribed to the stretching of double carbon bonds within the aromatic ring suggests partial decomposition of the cyclic structure over time.^48^ This trend is accompanied by the other C=C peak located at 1602 cm^−1^, mirroring the same reduction trend as the period of UV degradation increases.

**Fig. 4:**
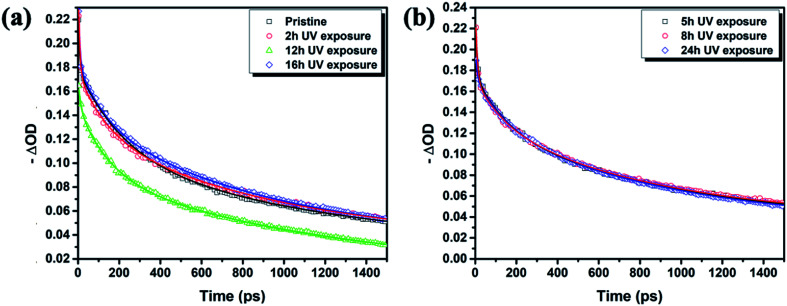
Transient band edge bleach kinetics (symbols) and the corresponding high order polynomial fits (lines) (a) for pristine, 2 h, 12 h, 16 h and (b) for 5 h, 8 h, 24 h ITO/PTAA/MAPI samples. The data are shown in two separate figures for the sake of presentation clarity.

After PTAA was subjected to UV light for a duration exceeding 2 h, another visible drop of signal intensity was observed in the area spanning from 1200 to 1380 cm^−1^. This region nests a well-defined peak population corresponding to the C–N stretching (1253, 1297 and 1317 cm^−1^)^49^ and further intensity drop of the amine bond fingerprint becomes noticeable after the 5–16 h window. The stretching of C–N bonds characteristic of aliphatic amines gives a weak signal at 1170 cm^−1^, seemingly unresponsive to UV illumination. Alternatively, this occurrence could be assigned to C–O etheric stretching caused by oxygen inclusion, in which case it would have been defined by a strong peak.^50^ Hence, we can dismiss this possibility, since peak intensity in our case is close to the background noise. The signal of the peak at 817 cm^−1^ arising from aromatic C–H bending and the corresponding shoulder assigned to the same bond within a meta xylene configuration become indistinguishable after the 12 h mark. It should be pointed out that the impact of UV degradation was insufficient to induce any morphological changes of PTAA films, as clearly indicated in AFM topography images (Fig. S2†). So far, we observed a certain degree of degradation in both perovskite and PTAA films; in order to pinpoint which of these structural transitions carries more weight on device performance it is necessary to take the charge transport mechanism into consideration. The first step in this direction is the estimation of hole injection efficiency from MAPI to PTAA, as this reveals the quality of the interface between the two. To this extent, we performed transient absorption spectroscopy experiments on fresh and UV pre-treated ITO/PTAA/MAPI bilayer devices. Fig. S6† depicts the resulting spectra of ΔOD as a function of wavelength at *t* = 0 ps for the pristine and UV degraded samples, following photoexcitation at 1026 nm with a pump fluence of 1.5 mJ cm^−2^. All spectra exhibit a main ΔOD peak at ∼750 nm which is attributed to the transient photo-induced bleaching of the band edge transition, while a photo-induced transient absorption in the range of 570–700 nm is additionally observed.^51^ In order to extract useful information regarding the charge carrier dynamics, time-resolved relaxation data were investigated by two well established models: three-exponential and high order polynomial fittings. From the first fitting procedure we extracted critical time components of the charge carrier transport processes occurring at the PTAA/MAPI interface. The latter fitting approach reveals the rates of recombination processes occurring within the perovskite films.^24,52^Fig. 4 displays the time decay fittings for studied samples based on the three-exponential function:1
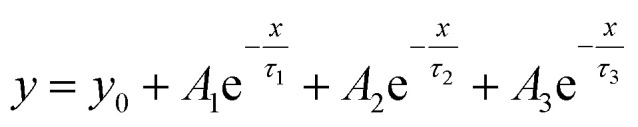
which allows the extraction of kinetic parameters (*τ*_1,_*τ*_2_ and *τ*_3_) summarized in Table 1.

**Table 1:** Characteristic time components for studied ITO/PTAA/MAPI architectures following exponential fitting (see the text)

UV exposure	*λ* (nm)	*τ*_1_ (ps)	*τ*_2_ (ps)	*τ*_3_ (ps)
Fresh	747	6	90	1328
2 h	747	7	143	1025
5 h	747	9	158	916
8 h	747	9	138	868
12 h	750	14	148	1263
16 h	747	7	124	905
24 h	749	8	143	890

In particular, the first time component (*τ*_1_) is attributed to charge carrier trapping at the perovskite grain boundaries and perovskite/HTL interfaces.^52,53^ It represents the transit time for excited carriers to move from the conduction band to the trap states. In principle, faster *τ*_1_ indicates quicker trap-filling, leading to larger splitting in the quasi-Fermi energy levels and consequently to more efficient free charge carrier injection, resulting in enhanced electrical characteristics of MAPI films. It should be mentioned that the density of trap states and their depth exert a noticeable effect on *τ*_1_ constant.^41^

**Fig. 5:**
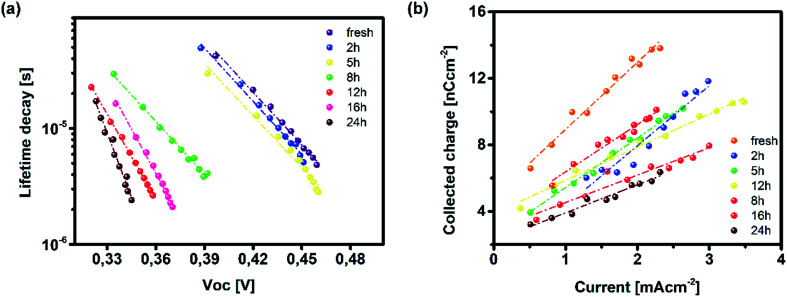
(a) Lifetime recombination decay as a function of background bias and (b) extracted charge from TPC measurements for fresh and degraded ITO/PTAA/MAPI bilayer devices.

Notably, the fresh sample exhibited slightly faster *τ*_1_ compared with the corresponding time components of UV degraded bilayers. The second time constant (*τ*_2_) represents the time required for hole injection from the perovskite layer into the PTAA polymer, with faster hole injection being indicative of better electrical characteristics of the devices. Inspection of values listed in Table 1 shows that the pristine sample had significantly faster *τ*_2_ in comparison to UV exposed samples. Interestingly the *τ*_2_ value doesn't vary considerably between different UV exposure times, implying that the PTAA/perovskite interface is stable and its charge transfer properties do not deteriorate beyond the initial degradation stage. Moreover, the third slow time component (*τ*_3_) which is representative of the exciton recombination time is the slowest in fresh films, meaning that the free carriers have longer availability for injection to the hole transporter. We performed additional fitting according to the polynomial model based on the following relation:2
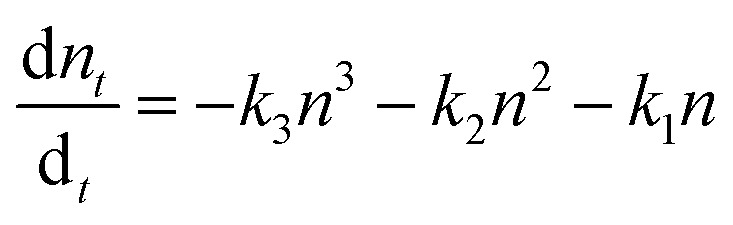
where *n* is the charge carrier density and *k*_1_, *k*_2_ and *k*_3_ are trap-assisted, bimolecular and Auger trimolecular recombination rates, respectively. All recombination rates for the studied samples are summarized in Tables 1 and S2.† A study by Wehrenfennig *et al.*^54^ highlights how slower bimolecular recombination rates (*k*_2_) are indicative of longer free-carrier diffusion length in the perovskite layer, which favours the efficiency of the planar heterojunction perovskite solar cells (PSCs). Notably, it becomes apparent that the pristine sample exhibits a slightly slower bimolecular recombination rate which agrees with the better electrical characteristics of the corresponding device. The charge carrier lifetime trend was probed using the TPV technique on devices having the previously defined photoactive bilayer structure, with the addition of a top electrode necessary for the formation of electrical contact (ITO/PTAA/MAPI/Ag). Experiments were performed under open circuit conditions and perturbations of small amplitude in order to ensure that the density of photogenerated charge carriers didn't interfere with the established electrical field. In this fashion, voltage transients followed single exponential decay and we were able to directly observe the lifetime of charge carriers. The resulting trend is depicted in Fig. 5a and it shows a clear segregation point set after 5 h of UV irradiation. Although comparable in values, the fresh specimen exhibited longer lifetimes than bilayers degraded for 2 and 5 h. The absence of a uniform lifetime recombination trend similar to the one observed in TAS measurements is linked to the effect of grain boundaries and loss of homogeneity within the bulk of bilayer constituents, both of which can be observed on a microsecond scale provided by TPV measurements.

**Fig. 6:**
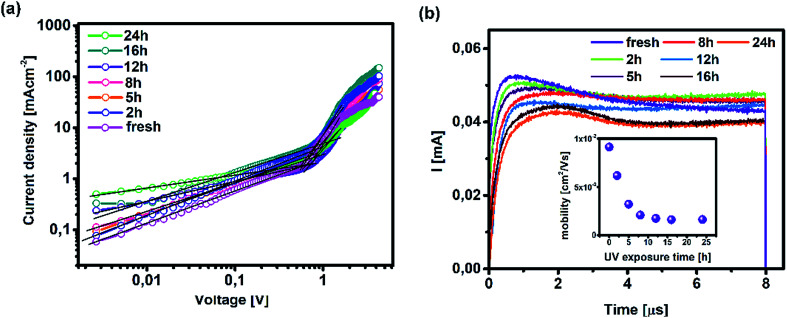
(a) Dark *J*–*V* current traces for hole-only devices (ITO/PTAA/MAPI/Au) upon UV exposure and (b) photo-CELIV transients of measured inverted planar solar cells (ITO/PTAA/MAPI/PC_60_BM/PFN/Ag) and corresponding mobilities (inset).

On the other hand, TAS was operated within the terahertz time-domain, which makes it non-sensitive to recombination phenomena occurring outside narrowly localized areas.^55^ Thus, we can correlate the sharp drop of carrier lifetime after the 5 h mark with the obstruction of charge flow through the sample arising from structural disordering, as well as capacitive effects introduced by the optoelectrical measurement protocol.^56,57^ Moreover, we note the absence of any apparent charge reservoir, as this occurrence would enhance the random-walk mechanism during the carrier transport and cause a decay trend of higher orders.^58,59^ As mentioned earlier, all transient decays in our case exhibited a mono-exponential trend which is not indicative of any effectual accumulation of charge carriers.^60^ The full set of TPV transients taken at each step of UV degradation is given in the ESI (Fig. S7†). The mechanism of charge extraction was probed using TPC experiments operating within the established framework of bilayer devices (ITO/PTAA/MAPI/Ag), and Fig. 5b illustrates the response from the reference and UV stressed samples after a 25 μs perturbation cycle. The extraction timeframe was chosen to overlap with the charge carrier lifetime to ensure that the measured response follows realistic operational circumstances, where a fraction of total charge within the device is lost due to the recombination process. It is evident that the fresh specimen exhibits the most effective charge collection properties, while damaged samples follow a distribution dictated by the extent of UV exposure. Such behaviour, in conjunction with the results obtained from TAS measurements, provides important insight into the mechanism of hole transfer through PTAA. As mentioned earlier, hole injection from MAPI to the polymer was effectively constant for all UV treated bilayers, indicating no successive degradation of transport across the interface. Hence, the drop of extraction efficiency observed by TPC analysis arises from the deterioration of electrical properties within the bulk of bilayer constituents. If we keep in mind the well-known fact that the mobility of a perovskite absorber far exceeds that of PTAA and other organic HTLs, this leads to the conclusion that the transient decay and resulting charge extraction trends are governed by the mobility of the PTAA film.^61–64^ Therefore, it is reasonable to pinpoint PTAA as an obstacle to efficient charge extraction, rather than the interface with MAPI. Interestingly, even though the previously discussed ATR results did not indicate any severe oxidative degradation of PTAA, it seems that structural alterations even under shorter UV irradiation noticeably affect its hole extraction capabilities. Considering that even smaller changes of the polymer structure result in such a response, it comes as no surprise why literature reports suggest doping of PTAA as an efficient way to improve performance stability of inverted planar solar cells.^17,65–67^ Photocurrent transients and corresponding temporal charge extraction at each stage of UV degradation are listed in the ESI.† An important step towards elucidation of the UV effect on the stability of PTAA and the light harvester is screening of defect states within the observed system. The mapping of defect states within observed bilayers was performed using SCLC measurements, upon addition of a gold electrode to ensure a suitable work function arrangement for the formation of hole-only ITO/PTAA/MAPI/Au devices. The measured SCLC response presented in Fig. 6a can be divided into three different regions for each of the resulting dark *J*–*V* curves. The first one corresponds to the ohmic response spread over the low-voltage range, where current follows linear dependency on the applied voltage. Afterwards, devices enter the trap-filled region marked by the abrupt increase of current, originating from the annihilation of defect population present within measured samples.^68^ Transition from ohmic governed to intermediate-voltage area allows extraction of trap densities (*n*_traps_), according to the following relation:^69^3
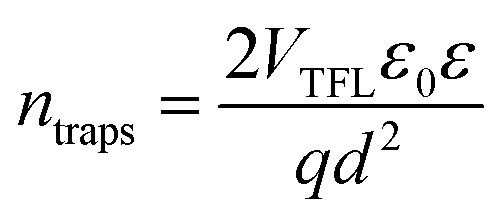
where *q* stands for elementary charge, *d* for thickness, while *ε* and *ε*_0_ are the dielectric constants of MAPI and vacuum permittivity, respectively. *V*_TFL_ represents the transition point between ohmic and trap-filled regimes and is defined as the trap filling limit voltage. Table 2 lists the calculated values for *n*_traps_ distribution in each of the tested devices. The initial increase of trapping states occurs after 2 h of UV treatment when the defect population saturates and stays levelled until the degradation time extends beyond 16 h, resulting in a further increase of trap concentration. The lowest population of traps was detected in the reference device, which is consistent with the results obtained from TAS, where the fresh specimen exhibited the fastest trap filling features. The increase of *n*_traps_ is accompanied by the shift of onset defining *V*_TFL_, which stems from the activation of additional trapping centres at higher voltages and reduction of film quality, as expected from devices subjected to prolonged degradation conditions.^70^ Moreover, the observed trend in the ohmic region suggests an increase of current corresponding to the leakage upturn, in accordance with UV exposure length.

**Table 2:** SCLC and photo-CELIV parameters as a function of UV degradation time for hole-only devices and complete planar inverted cells, respectively

UV exposure	*n*_traps_ (cm^−3^)	Mobility (cm^2^ V s^−1^)	*t*__1/2__/*t*__max__
Fresh	9.43 × 10^15^	9.13 × 10^−3^	3.35
2 h	1.19 × 10^16^	6.18 × 10^−3^	1.69
5 h	1.69 × 10^16^	3.22 × 10^−3^	1.60
8 h	1.81 × 10^16^	2.10 × 10^−3^	1.04
12 h	1.96 × 10^16^	1.76 × 10^−3^	0.86
16 h	2.67 × 10^16^	1.61 × 10^−3^	0.79
24	3.38 × 10^16^	1.64 × 10^−3^	0.91

Such behaviour is in line with that previously noted by TPC analysis and described reduction of PTAA charge extraction capabilities, since the formation of shunt pathways imposes a barrier for collection of photogenerated carriers.^71–73^ A further voltage increase leads to the space-charge-limited regime, a trap-free zone where device operation is governed only by the drift mobility of holes. The slope of the current–voltage curve in this domain is dictated by the quadratic growth (as per the Child–Langmuir law).^74^ In principle, it is possible to deduce hole mobility in the SCLC region according to the Mott–Gurney relation.^75^ However, in the case of absorbers with high intrinsic population of mobile species, like perovskites, straightforward estimation of drift mobility is convoluted, due to the unfavourable ratio between film thickness and size of the actual barrier for carrier injection.^76^ To that extent, we conducted photo-CELIV studies to gain insight into the charge carrier drift mobility distribution as a function of UV exposure. Since the photo-CELIV measurement protocol requires fully functional solar cells to reliably extract parameters necessary for estimation of drift carrier mobility, it was necessary to expand the active structure from a bilayer to a complete device (ITO/PTAA/MAPI/PC_60_BM/PFN/Ag). Thus, the existing bilayer structure was further expanded by deposition of a PCB_60_M layer on the reference and UV treated samples, followed by inclusion of PFN buffer and an Ag top electrode, resulting in the desired inverted planar architecture. We note that the interface established between MAPI and PC_60_BM formed in this way may differ from the case when they were to be formed prior to the UV exposure. Nonetheless, our aim was to monitor the capacity of PTAA for hole transport in response to UV degradation. Hence, exclusion of such an effect on the ETL and respective interface with the absorber helps prevent possible masking of the response. In principle, photo-CELIV utilizes short light pulses to generate charge carriers and extracts them afterwards, using a triangular ramp after a chosen delay time. The current response obtained in this way can be used to estimate the mobility of charge carriers according to the equation proposed by Lorrmann *et al.*:^77^4
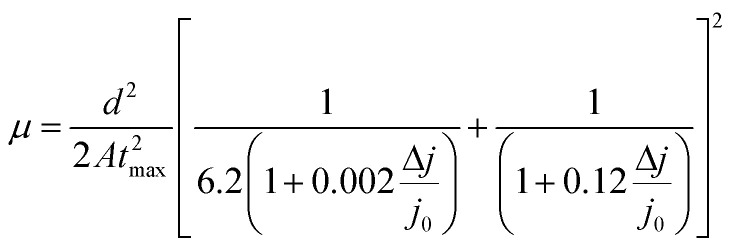
where *j*_0_ stands for displacement current, Δ*j* for current superimposed over *j*_0_, *d* for thickness, *A* is the extraction ramp and *t*_max_ is the time necessary for the current to reach its maximum value. Transient responses corresponding to all degradation scenarios presented in Fig. 6a clearly indicate the shift of the current peak following prolonged UV irradiation, caused by dispersive charge transport typically emanating from the increased defect population.^78^ Besides visual tracking, dispersion phenomena can be quantified using the ratio between *t*_max_ and the time defined by the full width at half maximum (*t*_1/2_) of the transient superimposed over displacement current. Charge transport devoid of any dispersive features corresponds to *t*_1/2_ : *t*_max_ = 1.2.^28^ Dispersive distribution of UV treated devices outlined in Table 2 shows an abrupt dip below 1.2 after 8 h of exposure, which serves as a clear indication of mobility dependence on the established electric field.^79^

It is evident from the inset of Fig. 6b how drift mobility of the charge carriers shows a high degree of sensitivity towards the influence of UV light even at shorter exposure periods, as 2 h degradation nearly halved the carrier drift velocity. After the 5 h mark, the recorded mobility was nearly three times lower than the reference value. Following this sharp decline, carrier mobilities of degraded devices settle at approximately one order of magnitude below that of their pristine counterpart. According to this trend, it is plausible to conclude that UV degradation of PTAA has a limited influence on the drift mobility of lead halide solar cells, under the assumption that the observed distribution is not asymmetrically influenced by the inclusion of the PC_60_BM layer. Nevertheless, since pristine PC_60_BM was used during fabrication of all devices, any potential trend disturbance introduced by the ETL would be equally exerted on all samples and the resulting distribution should remain preserved. Finally, readers should keep in mind that photo-CELIV detects collection of the fastest carriers at respective electrodes regardless of charge species sign, hence the difference between electrons and holes is imperceptible to this technique.

## Conclusions

In summary, we evaluated the effect of UV stress on the stability and charge transfer properties of PTAA and the interface formation with the MAPI absorber, through a series of rigorous optoelectrical characterization experiments. Each experimental procedure was conducted at different stages of UV exposure conjoined with the oxygen and humidity impact, to estimate the degree of degradation encountered under realistic operational circumstances. The resulting morphological degradation fingerprint points towards the significance of grain boundaries and ion migration, while the impact of UV exposure on the structural integrity of PTAA/MAPI bilayers exceeds that of oxygen and humidity. Furthermore, both the polymer and perovskite show only partial signs of degradation, lacking any indications of severe structural decomposition. Transient absorption spectroscopy revealed a stable PTAA/MAPI interface, resilient to UV intensity and extended periods of illumination. These findings coupled with TPC tracked charge extraction impose the conclusion that deterioration of PTAA film quality hinders efficient hole collection. The charge recombination decay indicates a non-uniform distribution of carrier lifetimes arising from impeded charge flow due to the structural disarrangement originating from the bulk of the bilayer, rather than the interface between the absorber and PTAA. Trap state distribution revealed by SCLC measurements demonstrates nearly monotonic UV induced dependency of defect population until the degradation threshold reaches the point of further trap generation at longer degradation times. This trend is accompanied by the formation of shut channels, allowing leakage of the current and appearance of a visible hysteretic response during the photoconversion process. The carrier drift mobility investigated using photo-CELIV revealed a noticeable initial drop upon UV exposure, followed by the relaxation to uniform values, suggesting partial dependence of the mobility on degradation time of PTAA. Additionally, tested samples exhibited signs of dispersive transport and increased dependence of drift mobility on the electric field, once the cells were subjected to extended periods of UV illumination. Finally, we can infer that PTAA degradation caused by UV light interferes with the mechanism of charge transport and appropriate actions should be taken to improve its operational resilience, such as doping with suitable materials or shielding from the adverse effects of ultraviolet irradiation by inclusion of an additional layer.

## Conflicts of interest

The authors declare no conflicts of interest.

## Supplementary Material

NA-001-C9NA00246D-s001
