# Supplementary material for: Limitations of a polymer-based hole transporting layer for application in planar inverted perovskite solar cells
Source: Nanoscale Adv. 2019 Jun 21;1(8):3107–18. doi: 10.1039/c9na00246d (PMC9417823; doi:10.1039/c9na00246d)
Supplement: NA-001-C9NA00246D-s001 [file NA-001-C9NA00246D-s001.pdf]

Electronic Supplementary Material (ESI) for Nanoscale Advances.
This journal is © The Royal Society of Chemistry 2019

Supplementary Information

Limitations of polymer-based hole transporting layer for application in planar inverted perovskite solar cells

Miloš Petrović,[a] Temur Maksudov,[a] Apostolos Panagiotopoulos,[ab] Efthymis Serpetzoglou,[cd] Ioannis Konidakis,[d] Minas M. Stylianakis,[a] Emmanuel Stratakis,[bd] Emmanuel Kymakis[*,a]

[a]*Department of Electrical & Computer Engineering, Hellenic Mediterranean University, Heraklion, 71410 Crete, Greece, Greece*

[b]*Department of Materials Science and Technology University of Crete Heraklion, 71003 Crete, Greece.*

[c]*Physics Department, University of Crete, 71003 Heraklion, Crete, Greece*

[d]*Institute of Electronic Structure and Laser (IESL), Foundation for Research and Technology−Hellas (FORTH), 71110 Heraklion, Crete, Greece.*

*Corresponding author:

Email: kymakis@staff.teicrete.gr

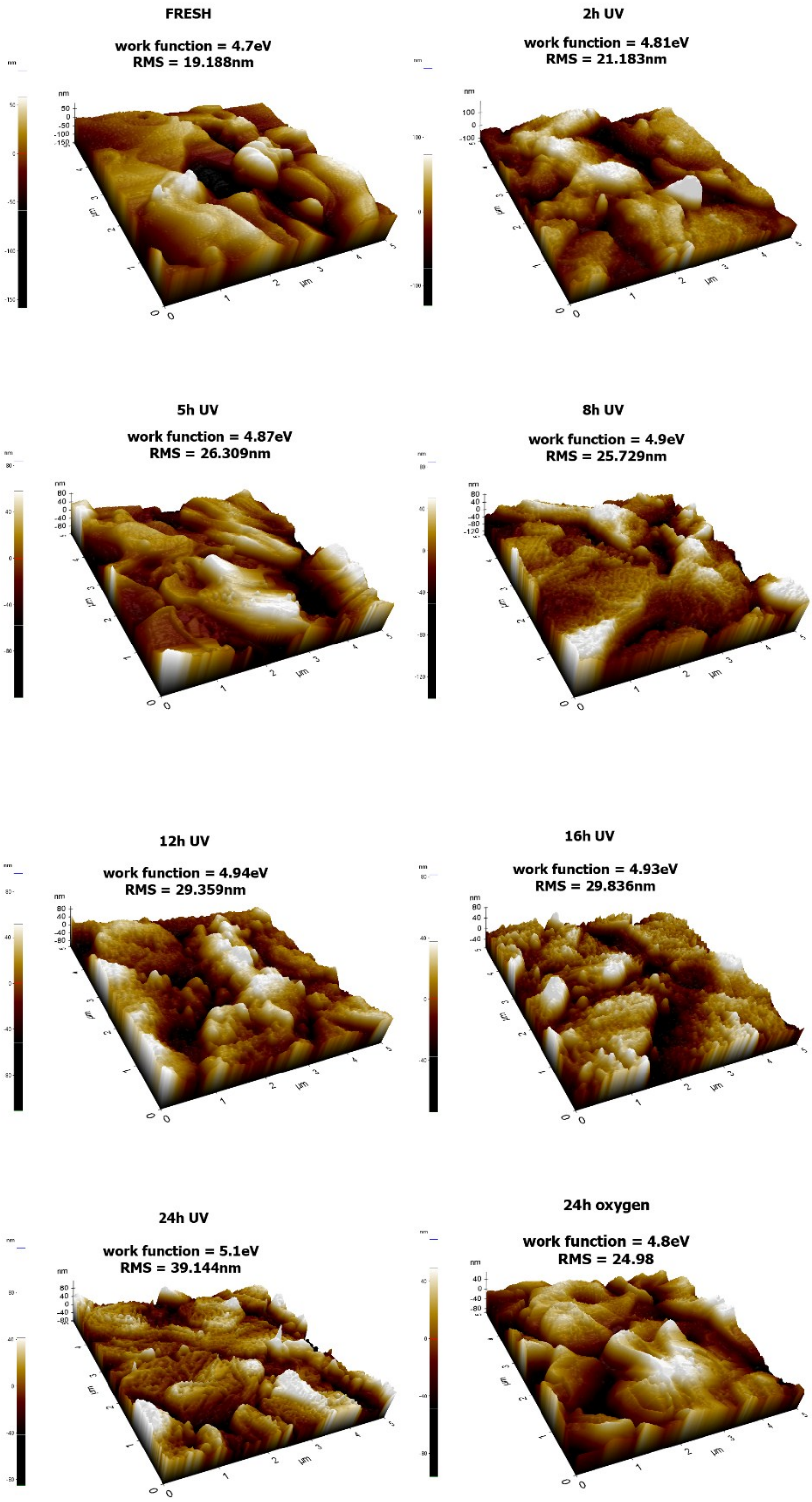

Fig. S1. AFM topography images of PTAA/MAPI bilayers resulting from various ageing conditions.

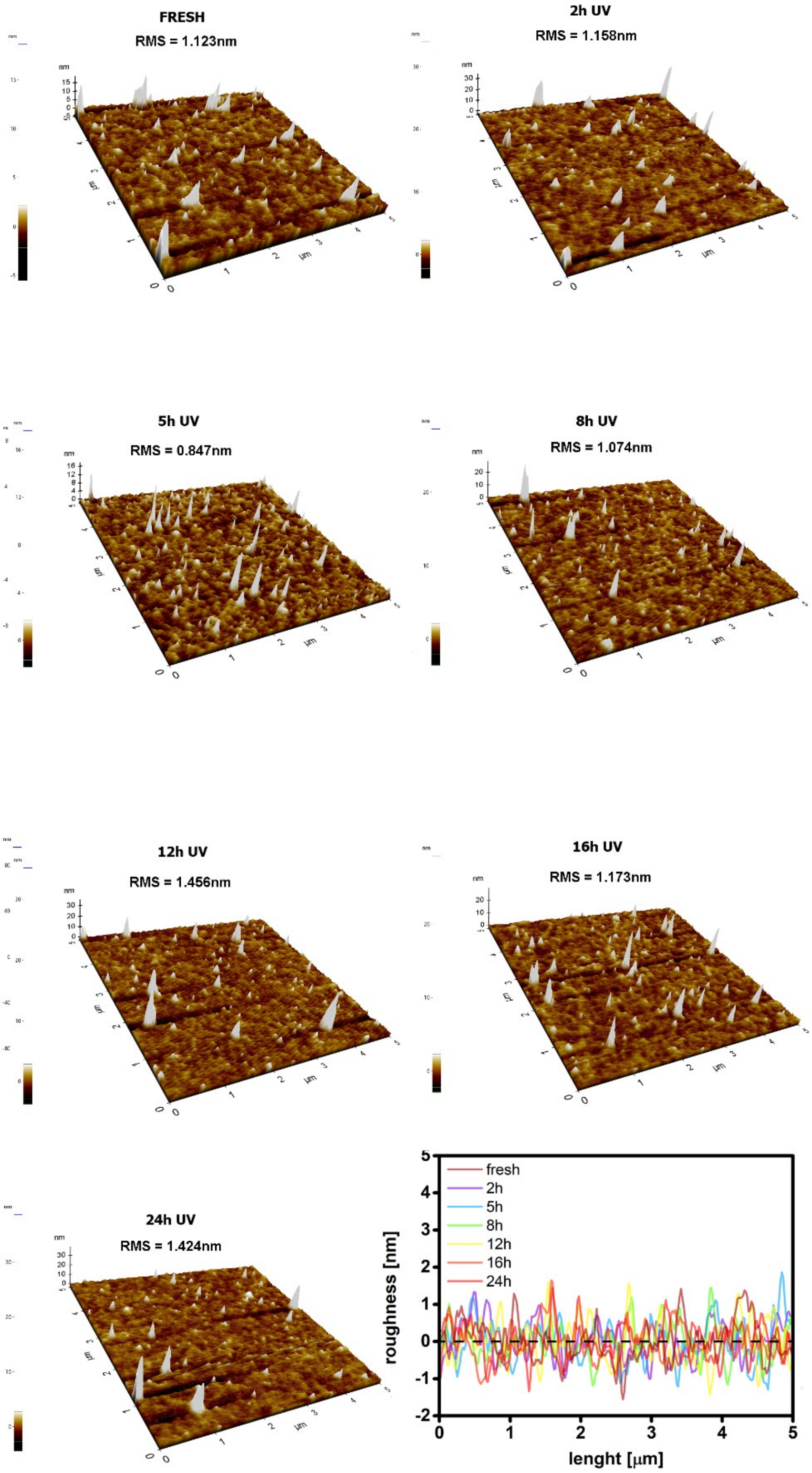

Fig. S2. AFM images of UV degraded PTAA films and corresponding roughness distribution.

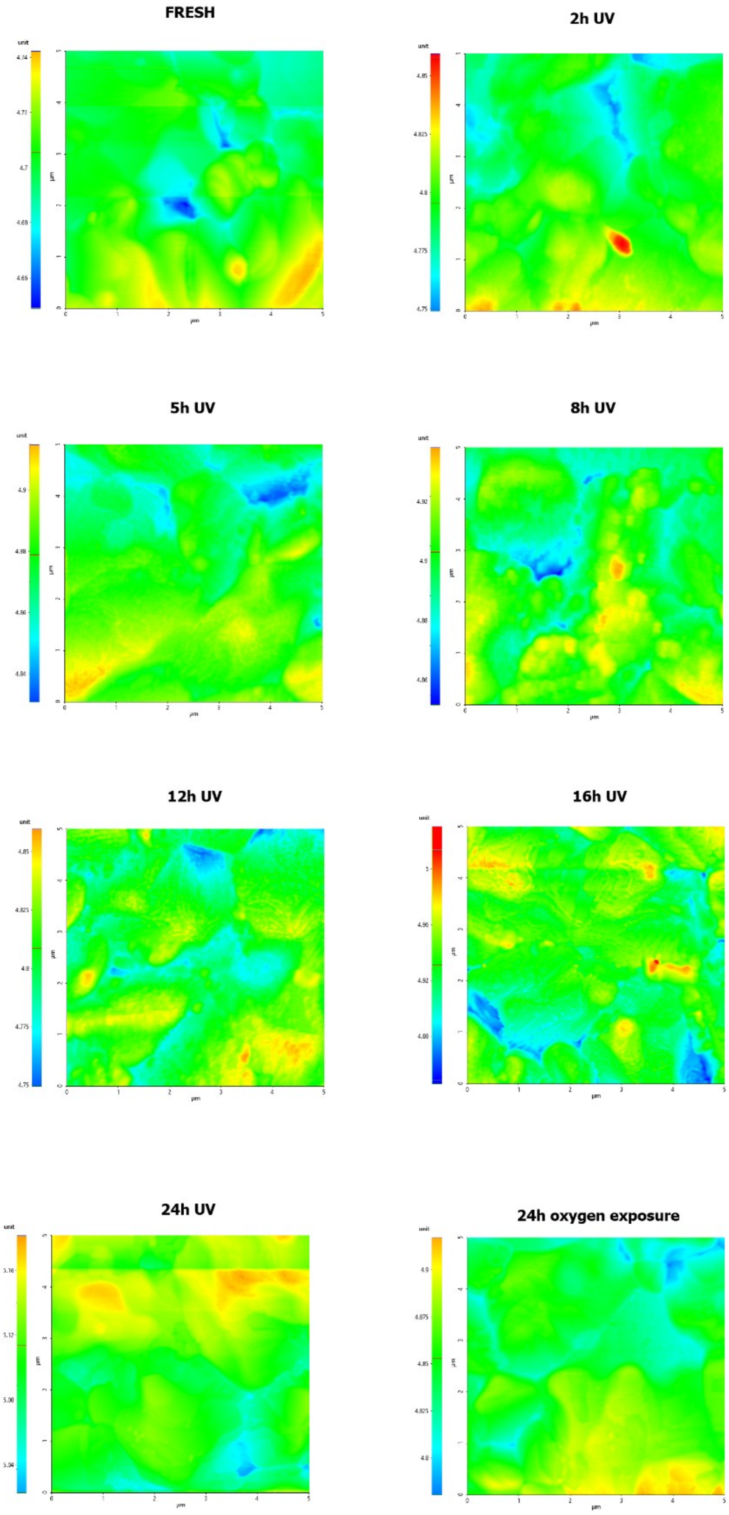

Fig. S3. Surface potential distribution of MAPI deposited over PTAA after various ageing periods and conditions.

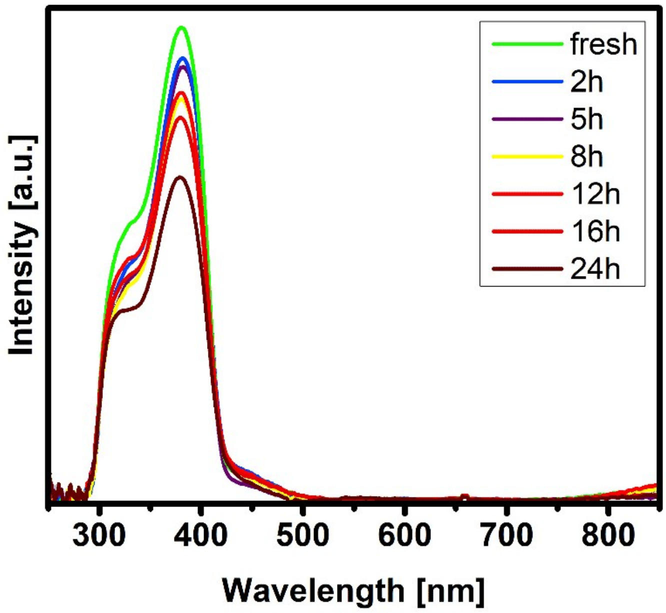

Fig. S4. UV-Vis spectra of neat and UV degraded PTTA films.

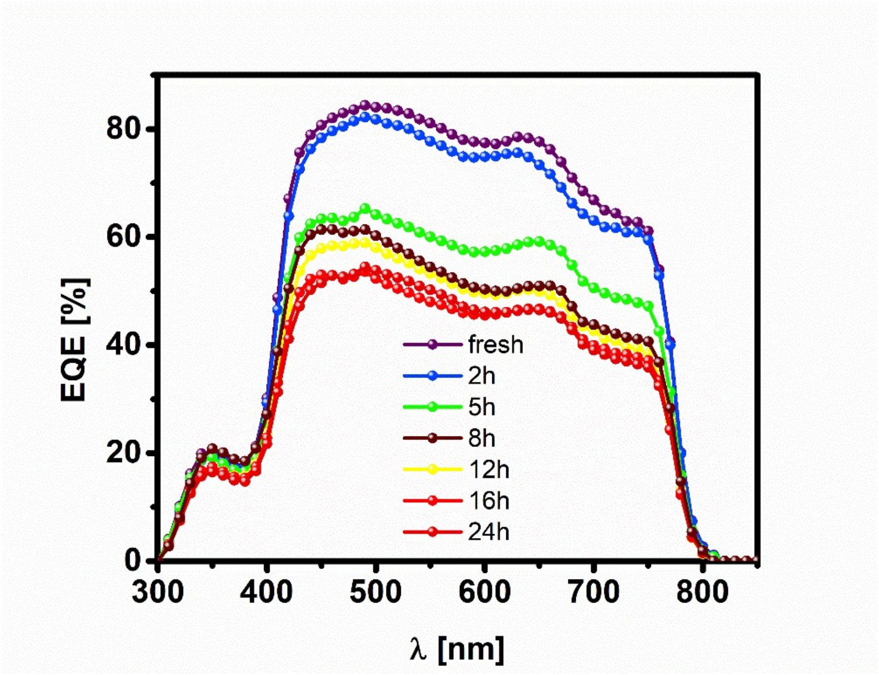

Fig. S5. External quantum efficiency of planar inverted perovskite solar cells (ITO/PTAA/MAPI/PCBM/PFN/Ag) subjected to various periods of UV exposure.

UV exposure	Voc [V]		Jsc [macm^{-2}]		FF [%]		PCE [%]	
	forward	*reverse*	*forward*	*reverse*	*forward*	*reverse*	*forward*	*reverse*
fresh	0,98	0,97	17,09	17,53	70,57	70,6	13,10	13,25
2h	0,99	0,99	16,53	16,23	67,11	68,59	12,12	12,18
5h	0,99	1,00	14,81	14,48	68,79	69,97	11,17	11,15
8h	1,01	1,01	15,69	15,31	63,79	65,9	11,14	11,18
12h	1,01	1,01	13,92	13,55	66,13	68,15	10,13	10,14
16h	0,99	0,99	13,59	13,21	59,53	61,23	8,80	8,87
24h	0,95	0,94	11,84	11,51	48,80	57,86	6,87	6,04

Table S1. Reduction in power conversion efficiency of planar inverted perovskite solar cells (ITO/PTAA/MAPI/$PC_{60}BM$/PFN/Ag) as a result of UV induced degradation.

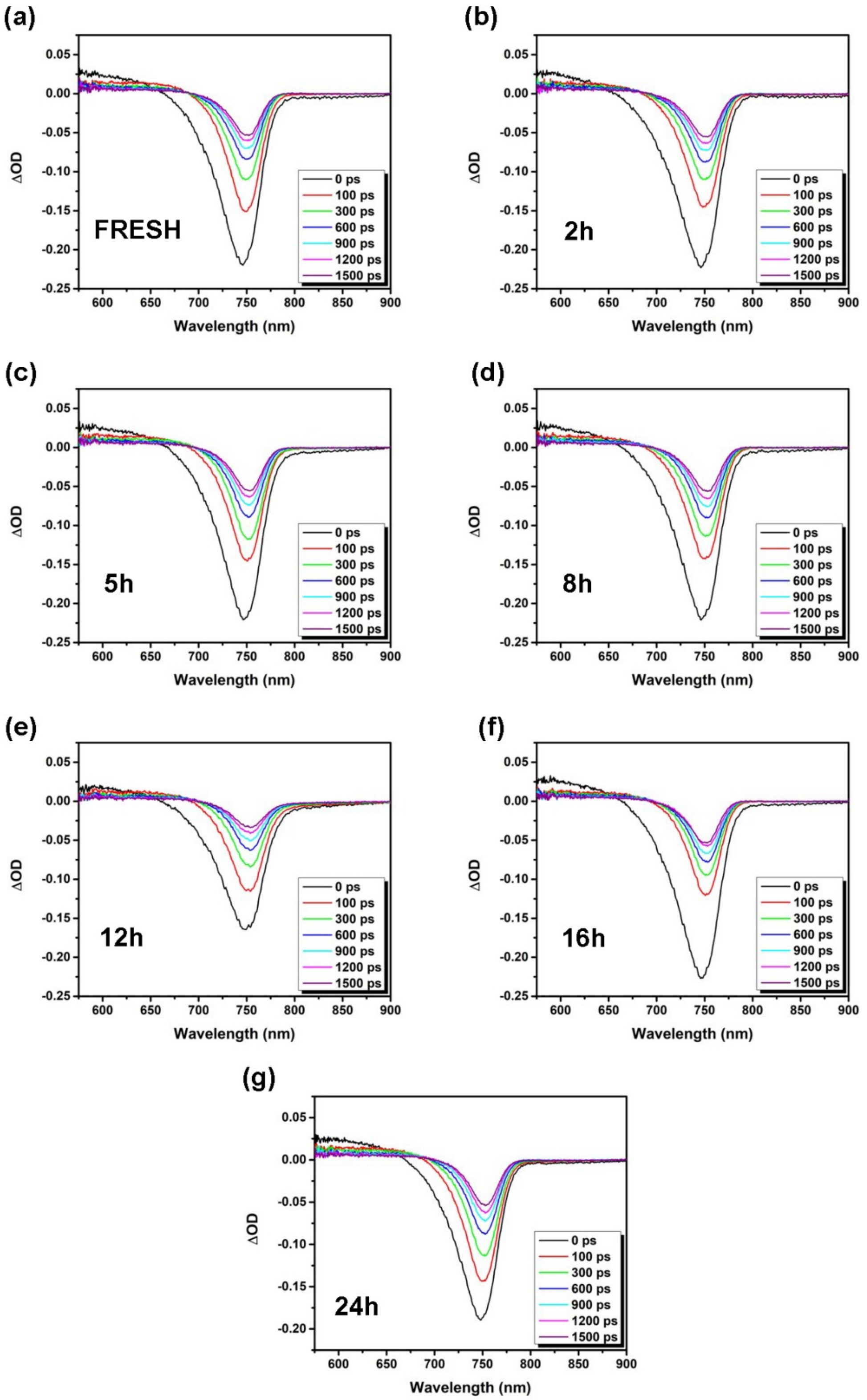

Fig. S6. Typical ΔOD vs wavelength graphs recorded by ultrafast TAS at various time delays for reference and UV degraded PTAA/MAPI bilayers.

UV exposure	*λ (nm)*	*k_3 (cm^6s^{-1}) ± 0.5*	*k_2 (cm^3s^{-1}) ± 0.3*	*k_1 (μs^{-1}) ± 0.1*
fresh	747	$5.7 \cdot 10^{-13}$	$9.8 \cdot 10^{-10}$	$8.6 \cdot 10^{-7}$
2h	747	$8.5 \cdot 10^{-13}$	$1.4 \cdot 10^{-9}$	$1.1 \cdot 10^{-6}$
5h	747	$7.6 \cdot 10^{-13}$	$1.3 \cdot 10^{-9}$	$1.0 \cdot 10^{-6}$
8h	747	$7.7 \cdot 10^{-13}$	$1.3 \cdot 10^{-9}$	$1.0 \cdot 10^{-6}$
12h	750	$6.5 \cdot 10^{-13}$	$1.1 \cdot 10^{-9}$	$1.0 \cdot 10^{-6}$
16h	747	$8.0 \cdot 10^{-13}$	$1.3 \cdot 10^{-9}$	$1.1 \cdot 10^{-6}$
24h	749	$8.3 \cdot 10^{-13}$	$1.8 \cdot 10^{-9}$	$1.7 \cdot 10^{-6}$

Table S2. Recombination rate constants for the studied ITO/PTAA/MAPI architectures fitted according to polynomial correlation.

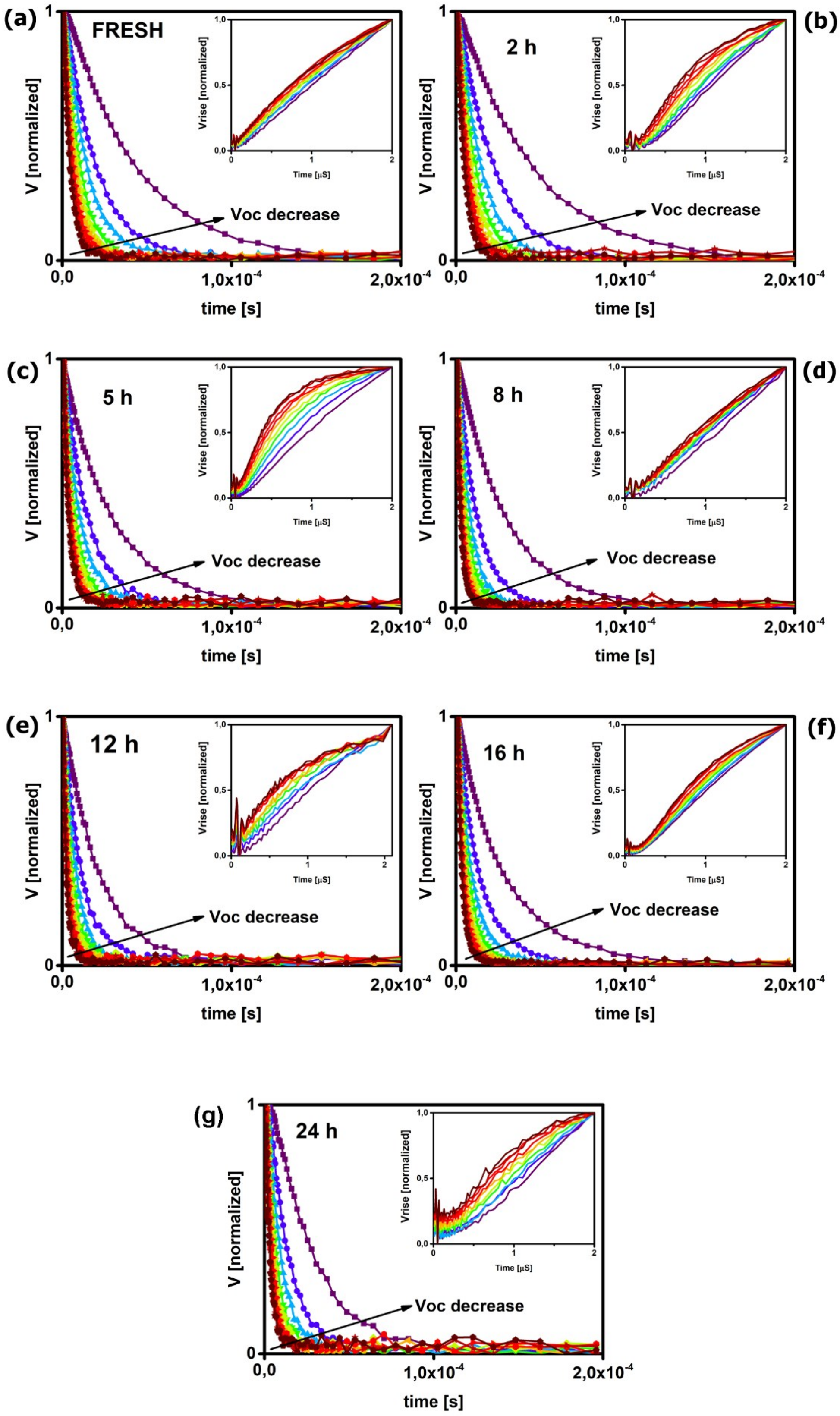

Fig. S7. Normalized small-perturbation transient photovoltage decay of ITO/PTAA/MAPI/Ag bilayer devices as a function of background bias recorded after UV stress and respective voltage rise responses (inset).

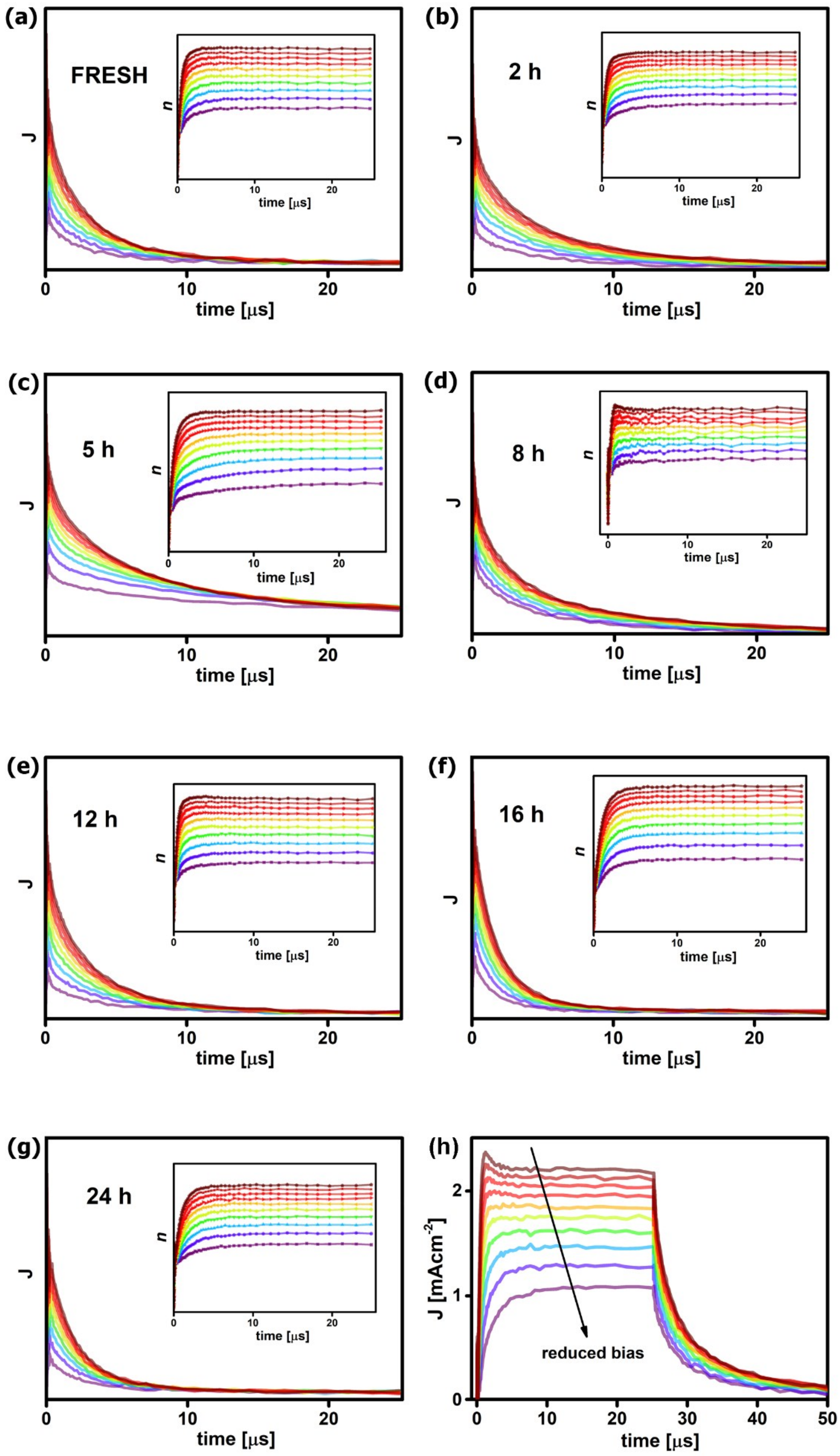

Fig. S8. (a-g) Photocurrent decay transients for bilayer devices at various UV exposure periods with respective collected charge over time (insets) and (h) absolute transient response of the fresh sample.

fresh		*2h*		*5h*		*8h*		*12h*		*16h*		*24h*	
Voc [V]	*lifetime [µs]*	*Voc [V]*	*lifetime [µs]*	*Voc [V]*	*lifetime [µs]*	*Voc [V]*	*lifetime [µs]*	*Voc [V]*	*lifetime [µs]*	*Voc [V]*	*lifetime [µs]*	*Voc [V]*	*lifetime [µs]*
0,40	42,23	0,39	49,28	0,39	29,75	0,33	29,49	0,32	22,69	0,34	16,38	0,32	17,15
0,42	21,50	0,41	23,87	0,42	12,80	0,35	15,26	0,33	11,36	0,35	8,39	0,33	12,32
0,43	15,41	0,42	15,87	0,44	8,44	0,36	10,19	0,34	8,41	0,35	6,19	0,33	9,23
0,44	11,24	0,43	12,27	0,44	6,49	0,37	7,83	0,34	6,16	0,36	4,77	0,33	8,02
0,44	9,45	0,44	10,10	0,45	5,33	0,38	6,52	0,35	5,04	0,36	3,88	0,33	5,93
0,45	7,81	0,44	8,51	0,45	4,46	0,38	5,41	0,35	4,26	0,36	3,33	0,34	4,70
0,45	6,73	0,44	7,44	0,46	3,79	0,38	5,54	0,35	3,73	0,37	2,89	0,34	3,83
0,45	6,19	0,45	7,31	0,46	3,43	0,39	4,44	0,35	3,28	0,37	2,57	0,34	3,26
0,46	5,58	0,45	5,85	0,46	2,99	0,39	3,83	0,36	2,93	0,37	2,28	0,34	2,87
0,46	4,85	0,45	5,11	0,46	2,83	0,39	4,10	0,36	2,65	0,37	2,10	0,35	2,41

Table S3. Charge recombination lifetimes extracted from PTAA/MAPI bilayer TPV transients as a function of background bias and UV exposure.

fresh		2h		5h		8h	
current [mAcm-2]	charge [nCcm-2]	current [mAcm-2]	charge [nCcm-2]	current [mAcm-2]	charge [nCcm-2]	current [mAcm-2]	charge [nCcm-2]
0,51	6,58	1,29	7,04	0,51	3,93	0,37	4,18
0,81	8,00	1,50	7,61	0,84	5,23	0,85	5,56
1,09	9,97	1,72	7,42	1,11	5,68	1,14	6,47
1,30	9,92	1,94	7,96	1,39	6,30	1,62	7,31
1,57	11,24	2,16	9,27	1,66	7,50	2,04	8,14
1,69	12,05	2,36	10,59	1,89	8,31	2,39	8,52
1,92	13,19	2,50	11,35	2,05	8,29	2,86	9,72
2,03	12,83	2,66	12,96	2,29	9,45	3,10	10,03
2,20	13,73	2,81	13,11	2,40	9,72	3,32	10,49
2,31	13,81	2,98	13,84	2,63	10,18	3,48	10,58

12h		16h		24h	
current [mAcm-2]	charge [nCcm-2]	current [mAcm-2]	charge [nCcm-2]	current [mAcm-2]	charge [nCcm-2]
0,82	4,27	0,59	3,49	0,51	3,22
1,11	5,26	0,92	4,41	0,81	3,60
1,45	6,16	1,28	4,89	1,09	3,83
1,58	6,39	1,58	6,39	1,30	4,75
1,80	6,52	1,86	5,90	1,57	4,68
1,95	6,75	2,19	6,69	1,69	4,87
1,95	7,08	2,44	6,60	1,92	5,58
2,12	7,34	2,60	7,07	2,03	5,66
2,17	7,39	2,77	7,22	2,20	5,81
2,26	7,77	3,00	7,95	2,31	6,36

Table S4. Charge extraction parameters calculated from TPC transient recorded at variable current bias and UV degraded PTAA/MAPI bilayers.
